# Numerical Analysis of Masticatory Forces on a Lower First Molar considering the Contact between Dental Tissues

**DOI:** 10.1155/2018/4196343

**Published:** 2018-04-10

**Authors:** Rosa Alicia Hernández-Vázquez, Beatriz Romero-Ángeles, Guillermo Urriolagoitia-Sosa, Juan Alejandro Vázquez-Feijoo, Ángel Javier Vázquez-López, Guillermo Urriolagoitia-Calderón

**Affiliations:** Instituto Politécnico Nacional, Escuela Superior de Ingeniería Mecánica y Eléctrica, Sección de Estudios de Posgrado e Investigación, Avenida Instituto Politécnico Nacional s/n, Edificio 5, 2do. Piso, Col. Lindavista, Unidad Profesional Adolfo López Mateos “Zacatenco”, 07320 Ciudad de México, Mexico

## Abstract

The aim of the present work is to identify the reactions of the dental organs to the different forces that occur during chewing and the transcendence of the union and contact maintained by the dental tissues. The study used a lower first molar biomodel with a real morphology and morphometry and consisting of the three dental tissues (enamel, dentin, and pulp) each with its mechanical properties. In it, two simulations were carried out, as would the process of chewing a food. One of the simulations considers the contact between the enamel and the dentin, and the other does not take it into account. The results obtained differ significantly between the simulations that consider contact and those that do not, establishing the importance of taking this contact into account. In this way, the theories that establish horizontal and lateral occlusion forces are present during the functional chewing process which are viable to be correct. The case studies carried out present not only the reasons for the failure of enamel but also the failure of the restoration materials used. This reflection will allow the development of more adequate materials, mechanical design of prostheses, implants, and treatment.

## 1. Introduction

The analysis of stress distribution in the dental organs under the action of occlusal loads entails a high complexity problem. This is due to the nonhomogeneity of various tissues that integrate their particular morphology, as each of these tissues has distinct mechanical and biological properties as well as a specific physiology, which constitutes a dynamic and specialized biomechanical system [1]. During mastication, the occlusal forces generated by the masticatory muscles, mainly the masseter, are applied to the dental organs, the enamel being the tissue that receives directly these loads. Dental enamel is a highly specialized organic tissue, made up of a complex crystalline structure. The hydroxyapatite prisms that compose it are ordered in the form of packages surrounded by organic matter, forming a mineralized matrix which gives it a property extremely hard but fragile [2]: that is, on its own, the enamel is extremely brittle; thus, it fractures easily. However, as it is supported by the dentine, the loads that arrive at the enamel are transmitted to this one, which, having an organic matrix greater than enamel support (type I collagen), gives it more elasticity. This situation improves the support of the normal loads transmitted by the enamel, allowing a better resistance to it.

**Figure 1:**
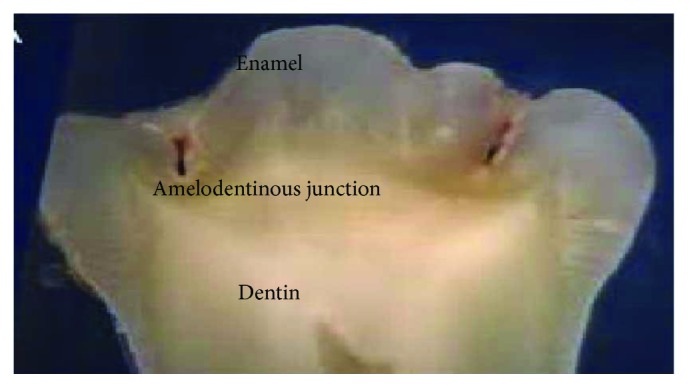
Sagittal section of a molar where the amelodentinous junction is observed.

The contact zone between both tissues, enamel and dentin, is not a smooth and regular area. It is described as an irregular scalloped boundary, where one can see protrusions of dentin projecting towards the enamel. Dentin and enamel are being formed by the same kind of cells (mineralized collagen and hydroxyapatite), so they are integrated. This causes an irregular border between both tissues [3]. And this established contact between the two tissues is called the amelodentinous junction (Figure 1).

This contact between tissues is very different from other joints that exist in the human body. Some authors consider it as a defined structure with a mechanical locking function linked to the function of the teeth [4]. Others describe it as an interface between two hard tissues with different matrix composition which gives them different physical properties [5]. There are other researchers who, on the contrary, maintain that it is a hybrid region that presents characteristics of both enamel and dentin [6]. Some more define it as a transition zone between enamel (prismatic) and dentin [7].

This intimate relation between the tissues, represented by the union of the amelodentinous junction, is of the utmost importance. Not only because of the symbiotic action that is established between them but also because of the clinical importance it represents. When the chewing process begins, the food is distributed throughout the occlusal face of the molars. As the food is crushed and transformed into the alimentary bolus, a dental contact occurs with the antagonist. This contact produces axial forces, which are transmitted from the enamel to the dentin and this leads them through the roots to be distributed along the periodontal ligament [8].

**Figure 2:**
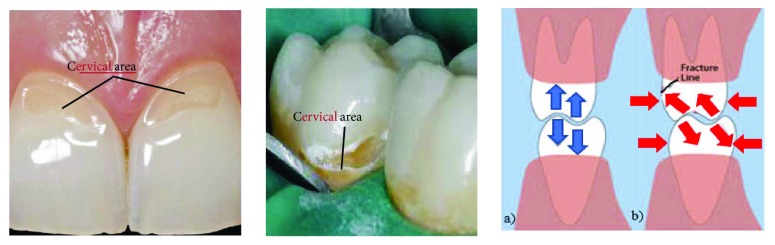
Fractures produced by the action of horizontal and lateral occlusal loads. (a) Normal forces acting during mastication. (b) Parafunctional forces.

Some theories suggest that horizontal and lateral occlusion forces (in the vestibulary-lingual direction) generated during mastication may also occur. But they are mainly due to unbalanced parafunctional and occlusal loads. This causes the tooth to flex, and tension and compression stresses are generated [9]. Causing a bow of the dental crown, in dentistry, it is said to take as *fulcrum* of the cervical region. Therefore, the dental organ opposes this load with an equal and opposite force, also generating a tension that manifests at itself as fatigue in the cervical area or cervical third [8]. These tensile stresses cause the hydroxyapatite prisms of the enamel to rupture, and a separation occurs between them (Figure 2).

The imbalance in the contact system between enamel and dentin can lead to dentin-enamel detachment and consequently to a possible fracture of the tooth [10, 11], and in the case of a restored tooth, it can lead to the failure of the obturation material. This phenomenon is called dental abfraction [12]. Contour analysis involving contact is of paramount importance for various areas of mechanical engineering, such as materials science and biomechanics.

It is now well known that the transmission of loads between the contact structures and the behavior of these interfaces significantly influence the response of the systems formed by the contact surfaces [13]. Thus, in the case of dentistry and the tissues that compose the dental organs, it is of utmost importance to carry out analyses of this type. The most recent studies have established that there are lesions produced not only by pathogens such as dental or traumatic caries (falls, bumps, pulp necrosis, etc.) but also by occlusion forces on the teeth. Such injuries involve the amelodentinous junction.

## 2. Materials and Methods

For this study, the first left lower molar of a 32-year-old male patient, apparently healthy, is used and an anatomical biomodel is made. For the acquisition of the molar images, 3D Imagenology was used. A digital volumetric tomography (DTV) of the maxilla and mandible with the cone-beam computed tomography (CBTC) system was used, from which DICOM 3D images are obtained. This system is used to obtain images of difficult-to-view tissues. It is widely used in medicine and dentistry in the craniofacial region. This new modality of Imagenology study offers accurate and high-quality three-dimensional representations of the bone elements present in the maxillofacial complex. Unlike the conventional tomography that shows consecutive cuts, the data obtained by a TVD and processed by the computer create a very good reconstruction of the studied volume.

In the tomographic study, a digital volumetric tomographer cone-beam (cone beam) brand Batex model EZ3D was used, which has a KvP of 90.0 mA and 3.8 light beam intensity. There were 477 images or cuts with a slide thickness of 0.5 mm. The space between the pixels (pixel spacing) has a ratio of 0.3/0.3 mm. The model obtained has high order elements (tetrahedral with a total of 8 nodes per element) and considers three different materials that correspond to the tissues conforming the dental organ (enamel, dentin, and pulp). The controlled discretization was carried out to obtain a total of 118,458 elements and 26,217 nodes. The mechanical properties of the tissues are described in Table 1.

For the simulation, the tissues are considered as materials that present a linear, elastic behaviour and whose internal structure is isotropic and homogeneous. As for the boundary conditions, since the roots of the molar are within the alveolus in the mandibular bone, the movement of the mandible is limited and controlled in this zone; therefore, the displacements and rotations are restricted in the directions of the *x*-, *y*-, and *z*-axes in the anatomical region corresponding to the roots of the molar.

During the chewing process, the jaw rises, and the lower molars encounter the upper ones and compress the food. It is what we properly called a bite. Subsequently, the jaw drops, and the molars are in contact and the bite force is no longer applied. To simulate this function, a series of charges were applied on the occlusal face of the molar and at the points of contact it establishes with its opposing piece (upper first molar) as described coming up.

**Figure 3:**
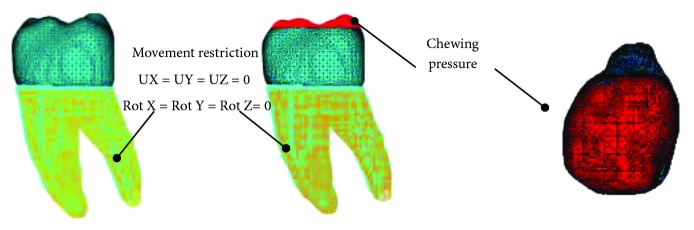
Boundary conditions and applied charges.

A load was applied to the aforementioned area, with magnitudes corresponding to the masticatory force (average functional occlusion) that occurs between both molars when the food is crushed (jaw elevation). Then, a load equal to zero is applied corresponding to the moment the jaw drops and so on. The magnitudes of the loads applied are shown in Table 2. These magnitudes are the ones that in several studies are described as those acting since the beginning of the masticatory process, which decrease as the food softens until it is ready for swallowing [14–18]. This is distributed locally in the application area in the form of a pressure (Figure 3).

To carry out the contact simulation, a coefficient of friction of 0.03 corresponding to the values established in the parameters of the ceramic-ceramic friction pairs [19–22] was used. The analyses were carried out using the finite element method.

## 3. Results and Discussion

The unitary strain, displacements, nominal stress, and *von Mises* stress were analysed during the application of loads and discharges to simulate the chewing process, obtaining the results in Tables 3–5.

**Figure 4:**
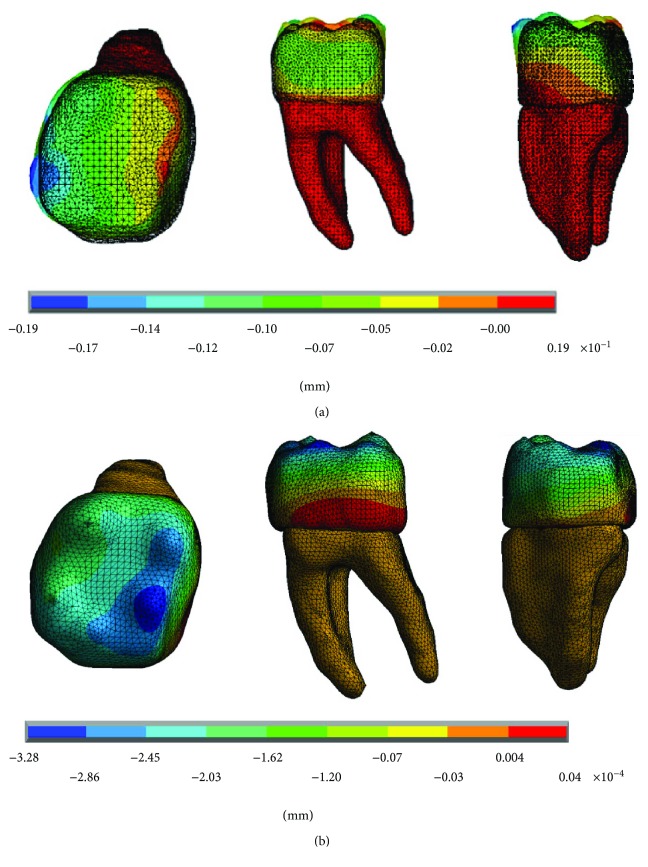
Displacements in the *x*-axis. (a) Noncontact. (b) Contact.

**Figure 5:**
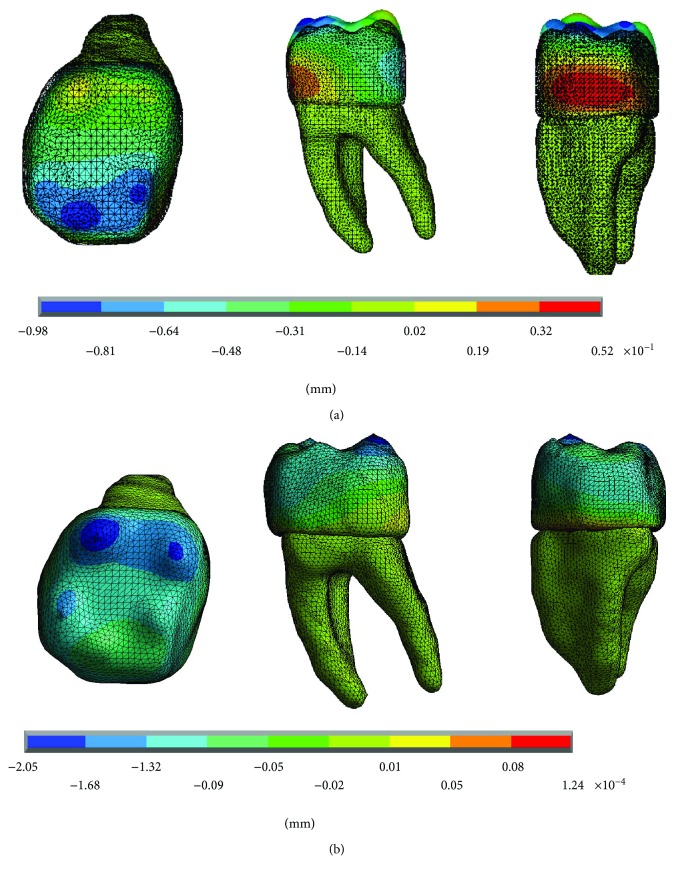
Displacements in the *y*-axis. (a) Noncontact. (b) Contact.

**Figure 6:**
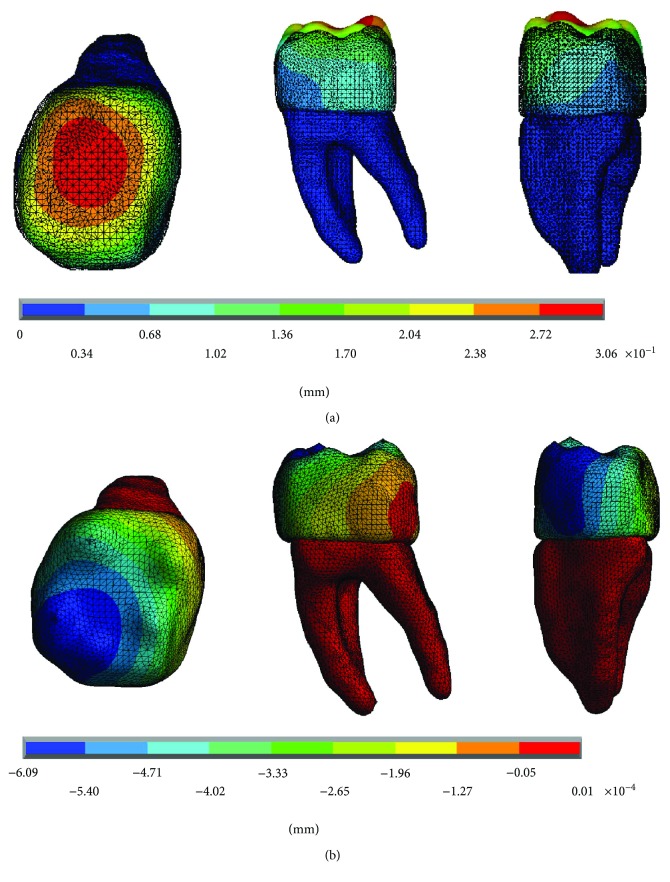
Displacements in the *z*-axis. (a) Noncontact. (b) Contact.

The results of the displacements for each of the axes, in the simulations with and without contact for the load of 150 N/mm^2^, present significant differences as shown in Figures 4–6.

**Figure 7:**
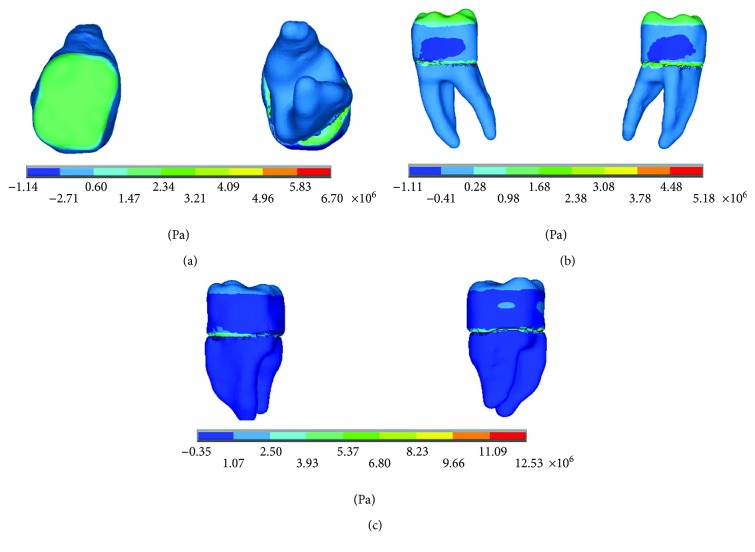
Nominal stress of the noncontact simulation. (a) *x*-axis. (b) *y*-axis. (c) *z*-axis.

**Figure 8:**
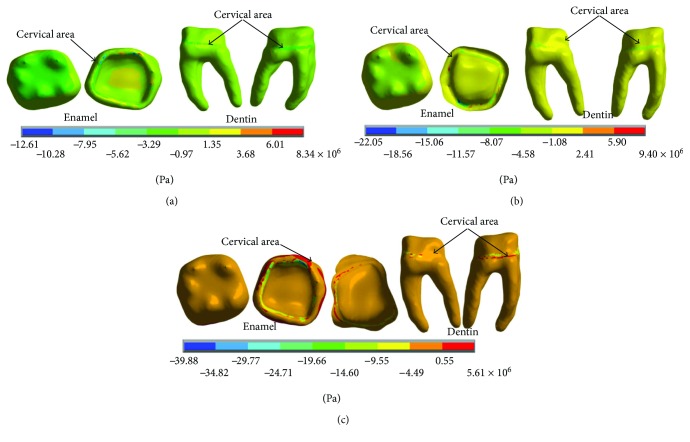
Nominal stress of the contact simulation. (a) *x*-axis. (b) *y*-axis. (c) *z*-axis.

The results of the nominal stresses for each of the axes, in the simulations with and without contact for the load of 150 N/mm^2^, present significant differences as shown in Figures 7 and 8.

In Figure 8, it is possible to observe the reactions in the internal structure of the tooth enamel and dentin, from the area under the enamel to the entire roots. In both cases, it is in the cervical zone specifically in the amelodentinous junction where the critical areas with the highest stress concentration are located.

**Figure 9:**
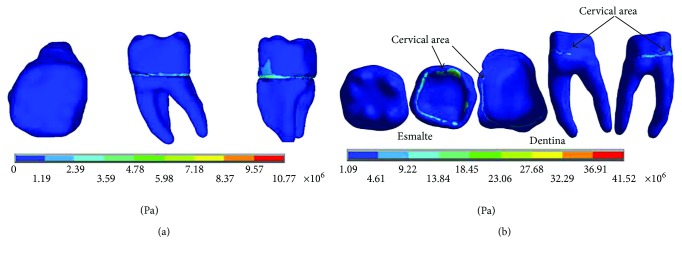
*von Mises* stress. (a) Contact. (b) Noncontact.

The results of the *von Mises* stresses for each of the axes, in the simulations with and without contact for the load of 150 N/mm^2^, present significant differences as shown in Figure 9.

**Figure 10:**
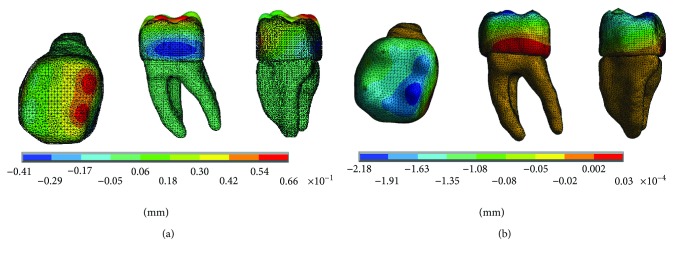
Displacements in the *x*-axis. (a) Noncontact. (b) Contact.

**Figure 11:**
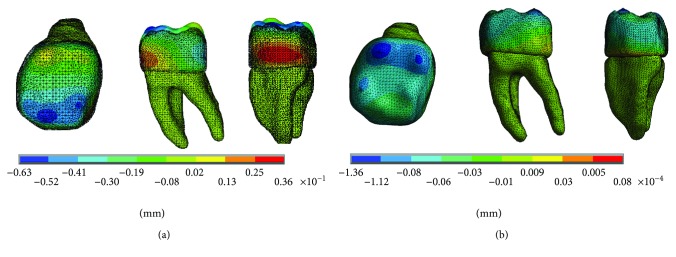
Displacements in the *y*-axis. (a) Noncontact. (b) Contact.

**Figure 12:**
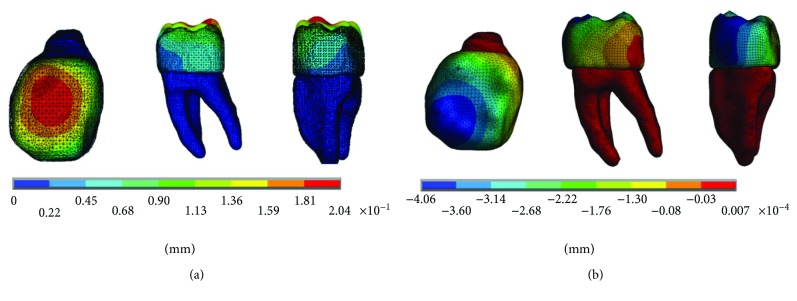
Displacements in the *z*-axis. (a) Noncontact. (b) Contact.

The results of the displacements for each of the axes, in the simulations with and without contact for the load of 100 N/mm^2^, present significant differences as shown in Figures 10–12.

**Figure 13:**
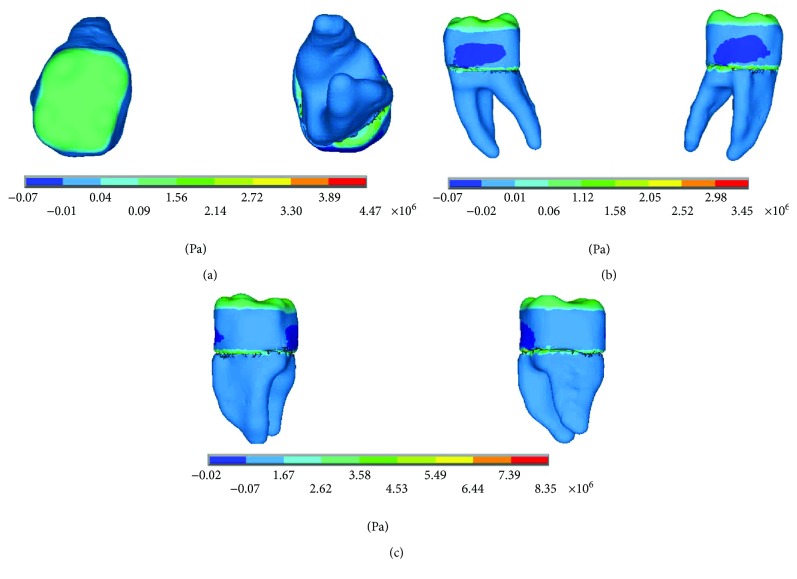
Nominal stress of the noncontact simulation. (a) *x*-axis. (b) *y*-axis. (c) *z*-axis.

**Figure 14:**
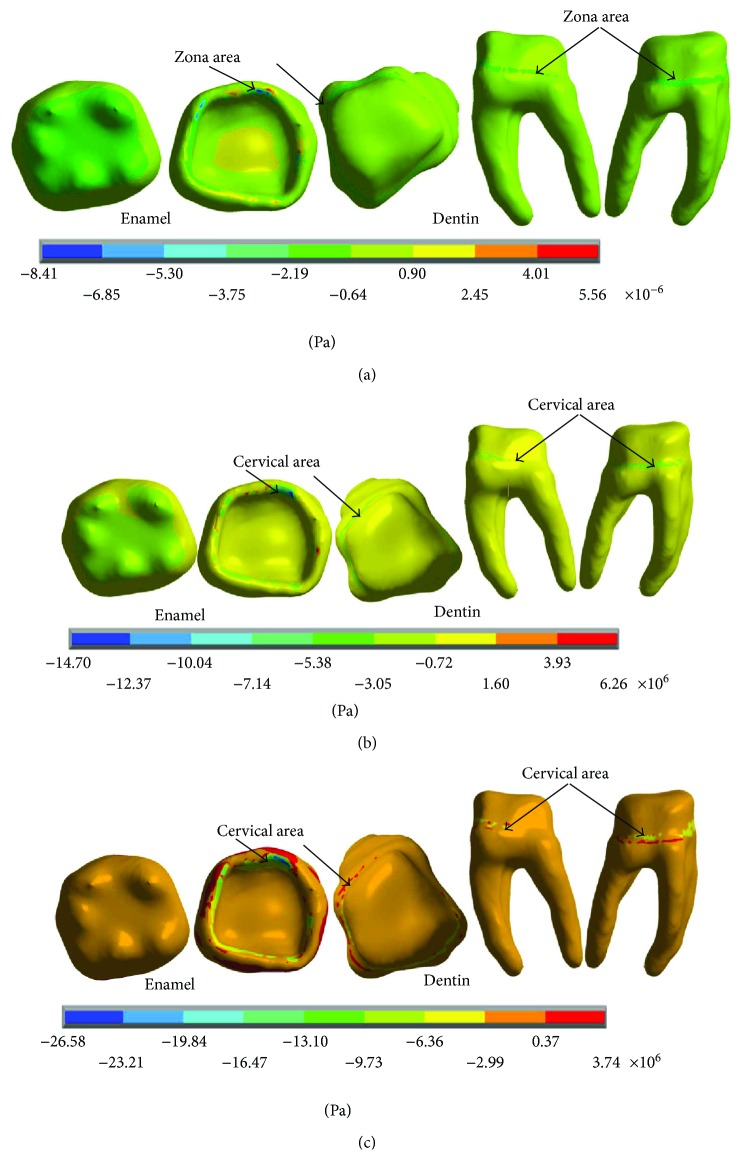
Nominal stress of the contact simulation. (a) *x*-axis. (b) *y*-axis. (c) *z*-axis.

The results of the nominal stresses for each of the axes, in the simulations with and without contact for the load of 100 N/mm^2^, present significant differences as shown in Figures 13 and 14.

**Figure 15:**
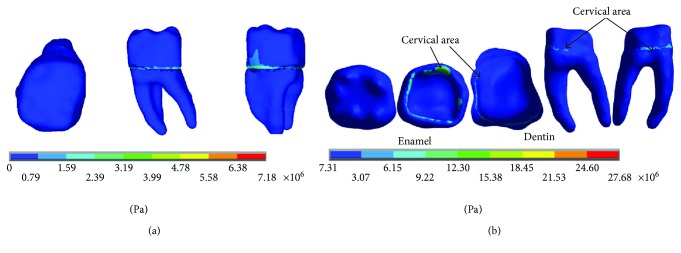
*von Mises* stress. (a) Contact. (b) Noncontact.

The results of the *von Mises* stresses for each of the axes, in the simulations with and without contact for the load of 100 N/mm^2^, present significant differences as shown in Figure 15.

**Figure 16:**
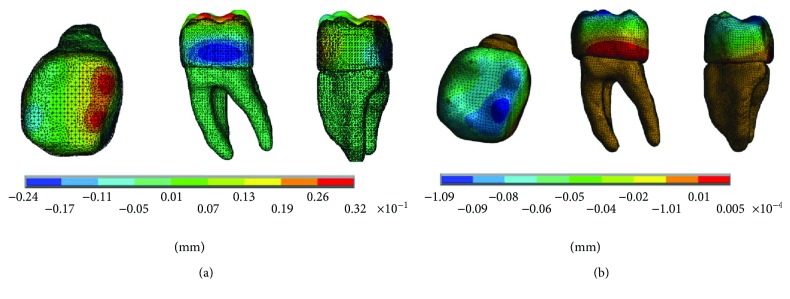
Displacements in the *x*-axis. (a) Noncontact. (b) Contact.

**Figure 17:**
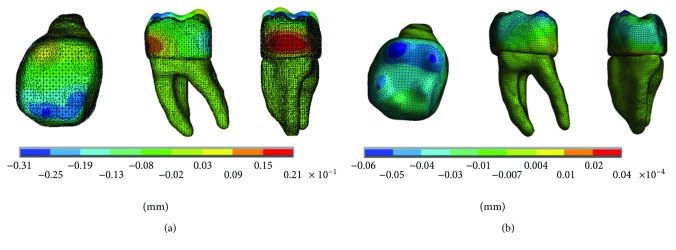
Displacements in the *y*-axis. (a) Noncontact. (b) Contact.

**Figure 18:**
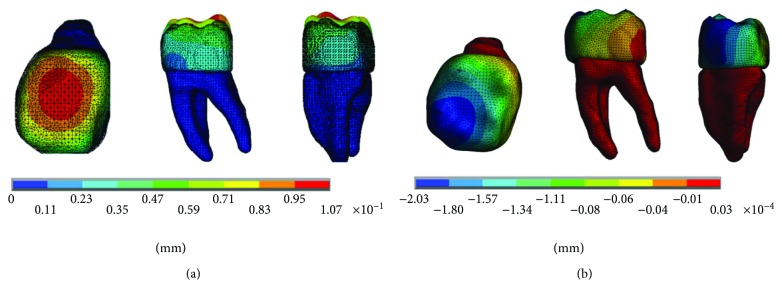
Displacements in the *z*-axis. (a) Noncontact. (b) Contact.

The results of the displacements for each of the axes, in the simulations with and without contact for the load of 50 N/mm^2^, present significant differences as shown in Figures 16–18.

**Figure 19:**
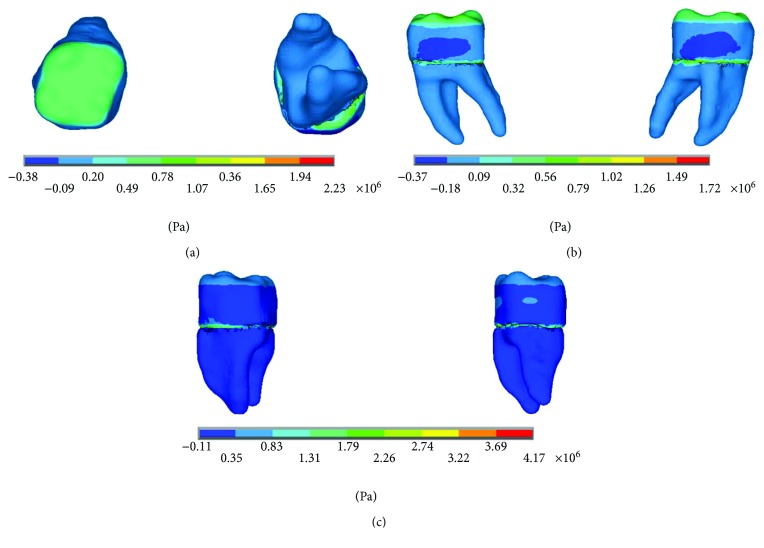
Nominal stress of the noncontact simulation. (a) *x*-axis. (b) *y*-axis. (c) *z*-axis.

**Figure 20:**
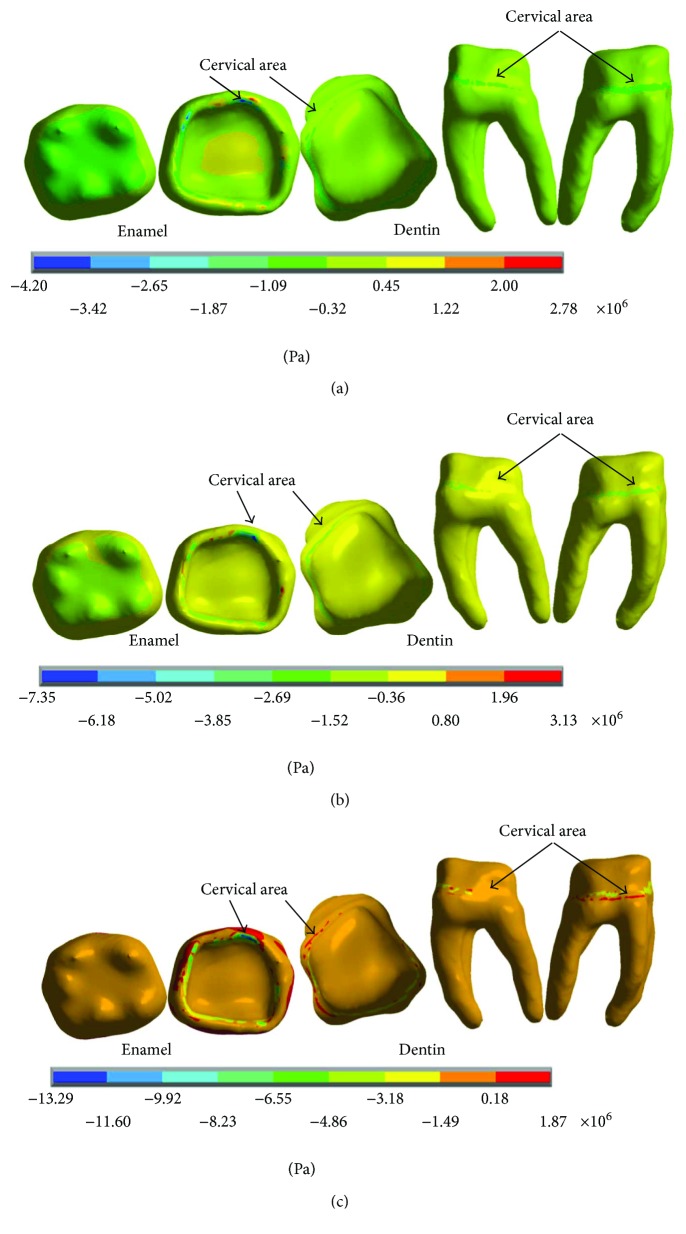
Nominal stress of the contact simulation. (a) *x*-axis. (b) *y*-axis. (c) *z*-axis.

The results of the nominal stresses for each of the axes, in the simulations with and without contact for the load of 50 N/mm^2^, present significant differences as shown in Figures 19 and 20.

**Figure 21:**
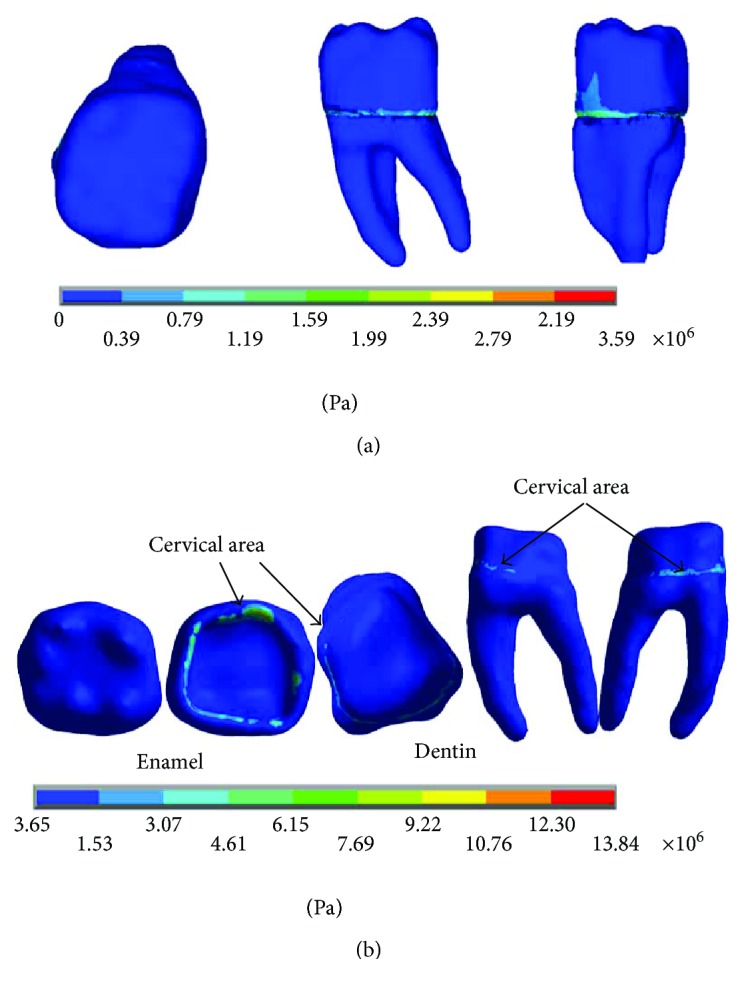
*von Mises* stress. (a) Contact. (b) Noncontact.

The results of the *von Mises* stresses for each of the axes, in the simulations with and without contact for the load of 50 N/mm^2^, present significant differences as shown in Figure 21.

The results obtained differ significantly between the simulations that consider contact and those that do not. In the case of those who did not consider it, such discrepancies are possible because each tissue, enamel and dentin, reacts independently and not as a biological system in synergy. The loads are received by the enamel and reach the dentin. However, in the situations in which contact is considered, although the model states that they are two different materials and with specific mechanical properties for each one, they work like a system as a whole, as it nearly happens. As mentioned before, the amelodentinous junction is not a clear border, as there are interdigitations and extensions between both tissues, a situation that is very difficult to simulate accurately.

On the other hand, thanks to the union of dentin and enamel that is established in the biomodel; it is possible to observe that horizontal and lateral occlusion forces occur during the functional chewing process, and these produce displacements in the dental enamel at the vestibulary-lingual direction, creasing stress generation and compression. This can be observed, especially in the reactions that occur on the *z*-axis in all cases. So it is possible that the mentioned bowing phenomenon of the dental organ occurs. Although the applied loads do not lead to fatigue and the consequent failure of the enamel, the results show that, in fact, in the cervical zone, a flexor moment is generated making the unit deformations the most critical ones.

The dental organ opposes this load with an equal opposite direction force generating a tension that manifests itself as a kind of fatigue in the cervical third. In the figures where it is possible to observe separately the dental crown constituted by enamel and the structure that conforms the internal part and the roots of the dental organ, it is there confirmed that the deformations and maximum stress are presented in cervical right, in the amelodentinous junction.

These clinical results are of great importance because they make it appear that the treatment plans and the materials used for the restoration of the dental organs are questioned. Throughout the life of the person, the dental organs will be subjected to cyclic chewing charges. External factors such as psychological stress or systemic diseases can alter the balance of masticatory functioning. Usually when this happens, homeostatic factors regulate and establish the balance of the masticatory process, but sometimes this does not happen. Therefore, the present work establishes the bases for the analysis of the reactions of the dental organs, mainly in the critical zones found as it is the cervical zone. It serves as a precedent for the mechanical phenomena that occur in this area. It is necessary to deepen these analyses that will allow the paradigms change in terms of the treatments and restoration materials that are currently used.

## 4. Conclusions

The case studies carried out within this work consider not only the reasons for the failure of enamel but also the possible failure of the restoration materials used. It raises the possibility of questioning dental materials and the way they are used today. It is important to analyse the transcendence and nature of the relationship that enamel and dentine establish with their particular mechanical properties, the first was highly rigid but fragile and the other was not so hard but more elastic. This contact relationship between them allows the unique operation of the teeth. Rather than thinking on restorative materials of high hardness, it would be convenient to think on how to restore the dental organs considering this symbiotic relationship between them in order to ensure a functioning that, without affecting the opposing teeth and supporting bone, it kept with the protection to the dental pulp and thereby be able to restore the function as close to the original biological.

It is important to emphasize the transcendence of the use of high fidelity biomodels as the one used in this work. The biomodels consider the real morphology and morphometry for the tissues that conform the structures with their specific mechanical properties, and they adequately characterize them, allowing results of greater attachment to reality. Finally, it is important to mention that most studies of loads on molars are performed on biomodels which lack precision and do not consider the contact between the tissues (amelodentinous junction), hence the transcendence of the present work.

## Figures and Tables

**Table 1:** Mechanical properties used in the analysis.

Dental tissue	*Young's* module	Dimensional *Poisson* ratio	Density
Enamel	70 GPa	0.30	0.25 g/cm^3^
Dentin	18.3 GPa	0.30	0.31 g/cm^3^
Pulp	2 GPa	0.45	0.1 g/cm^3^

**Table 2:** Magnitudes of applied loads.

Número de paso de carga	Carga en N/mm^2^
1	150
2	0
3	100
4	0
5	50
6	0

**Table 3:** Simulation results for a load of 150 N/mm^2^.

Unitary strain: 0.003571			Unitary strain: 0.000036602	
	Contact		Noncontact	
	Maximum	Minimum	Maximum	Minimum
Displacements X	1.04 × 10^−1^ mm	−0.59 × 10^−1^ mm	−3.28 × 10^−4^ mm	0.04 × 10^−4^ mm
Displacements Y	−0.98 × 10^−1^ mm	0.52 × 10^−1^ mm	−2.05 × 10^−4^ mm	1.24 × 10^−4^ mm
Displacements Z	3.06 × 10^−1^ mm	0 mm	−6.09 × 10^−4^ mm	0.01 × 10^−4^ mm
Nominal stress X	6.70 × 10^6^ Pa	−1.14 × 10^6^ Pa	−12. 61 × 10^6^ Pa	8.34 × 10^6^ Pa
Nominal stress Y	5.18 × 10^6^ Pa	−1.11 × 10^6^ Pa	−22.05 × 10^6^ Pa	9.40 × 10^6^ Pa
Nominal stress Z	12.53 × 10^6^ Pa	−0.35 × 10^6^ Pa	−39.88 × 10^6^ Pa	5.61 × 10^6^ Pa
*von Mises* stress	10.77 × 10^6^ Pa	0 Pa	41.52 × 10^6^ Pa	1.09 × 10^6^ Pa

**Table 4:** Simulation results for a load of 100 N/mm^2^.

Unitary strain: 0.003571			Unitary strain: 0.000036602	
	Contact		Noncontact	
	Maximum	Minimum	Maximum	Minimum
Displacements X	0.66 × 10^−1^ mm	−0.41 × 10^−1^ mm	−2.18 × 10^−4^ mm	0.03 × 10^−4^ mm
Displacements Y	−0.63 × 10^−1^ mm	0.36 × 10^−1^ mm	−1.36 × 10^−4^ mm	0.08 × 10^−4^ mm
Displacements Z	2.04 × 10^−1^ mm	0 mm	−4.06 × 10^−4^ mm	0.007 × 10^−4^ mm
Nominal stress X	4.47 × 10^6^ Pa	−0.07 × 10^6^ Pa	−8. 41 × 10^6^ Pa	5.56 × 10^6^ Pa
Nominal stress Y	3.45 × 10^6^ Pa	−0.07 × 10^6^ Pa	−14.70 × 10^6^ Pa	6.26 × 10^6^ Pa
Nominal stress Z	8.35 × 10^6^ Pa	−0.02 × 10^6^ Pa	−26.58 × 10^6^ Pa	3.74 × 10^6^ Pa
*von Mises* stress	7.18 × 10^6^ Pa	0 Pa	27.68 × 10^6^ Pa	7.31 × 10^6^ Pa

**Table 5:** Simulation results for a load of 50 N/mm^2^.

Unitary strain: 0.003571			Unitary strain: 0.000036602	
	Contact		Noncontact	
	Maximum	Minimum	Maximum	Minimum
Displacements X	0.32 × 10^−1^ mm	−0.24 × 10^−1^ mm	−1.09 × 10^−4^ mm	0.005 × 10^−4^ mm
Displacements Y	−0.31 × 10^−1^ mm	0.21 × 10^−1^ mm	−0.06 × 10^−4^ mm	0.04 × 10^−4^ mm
Displacements Z	1.07 × 10^−1^ mm	0 mm	−2.03 × 10^−4^ mm	0.03 × 10^−4^ mm
Nominal stress X	2.23 × 10^6^ Pa	−0.38 × 10^6^ Pa	−4.20 × 10^6^ Pa	2.78 × 10^6^ Pa
Nominal stress Y	1.72 × 10^6^ Pa	−0.37 × 10^6^ Pa	−7.35 × 10^6^ Pa	3.13 × 10^6^ Pa
Nominal stress Z	4.17 × 10^6^ Pa	−0.11 × 10^6^ Pa	−13.29 × 10^6^ Pa	1.87 × 10^6^ Pa
*von Mises* stress	3.1 × 10^6^ Pa	0 Pa	13.84 × 10^6^ Pa	3.65 × 10^6^ Pa
